# Innocuous, Highly Conductive, and Affordable Thermal Interface Material with Copper-Based Multi-Dimensional Filler Design

**DOI:** 10.3390/biom11020132

**Published:** 2021-01-20

**Authors:** Woochang Kim, Chihyun Kim, Wonseok Lee, Jinsung Park, Duckjong Kim

**Affiliations:** 1 Department of Applied Nano Mechanics, Korea Institute of Machinery and Materials, 156 Gajeongbuk-ro, Daejeon 34103, Korea; chang1121@korea.ac.kr (W.K.); kimeoup@korea.ac.kr (C.K.); 2 Department of Control and Instrumentation Engineering, Korea University, 2511 Sejong-ro, Sejong 30019, Korea; lws729@gmail.com; 3 Department of Mechanical Engineering, Gyeongsang National University, 501 Jinju-daero, Jinju, Gyeongnam 52828, Korea

**Keywords:** nano safety, thermal interface material, copper nanoparticle, multi-dimensional filler, high thermal conductivity, low cost

## Abstract

Thermal interface materials (TIMs), typically composed of a polymer matrix with good wetting properties and thermally conductive fillers, are applied to the interfaces of mating components to reduce the interfacial thermal resistance. As a filler material, silver has been extensively studied because of its high intrinsic thermal conductivity. However, the high cost of silver and its toxicity has hindered the wide application of silver-based TIMs. Copper is an earth-abundant element and essential micronutrient for humans. In this paper, we present a copper-based multi-dimensional filler composed of three-dimensional microscale copper flakes, one-dimensional multi-walled carbon nanotubes (MWCNTs), and zero-dimensional copper nanoparticles (Cu NPs) to create a safe and low-cost TIM with a high thermal conductivity. Cu NPs synthesized by microwave irradiation of a precursor solution were bound to MWCNTs and mixed with copper flakes and polyimide matrix to obtain a TIM paste, which was stable even in a high-temperature environment. The cross-plane thermal conductivity of the copper-based TIM was 36 W/m/K. Owing to its high thermal conductivity and low cost, the copper-based TIM could be an industrially useful heat-dissipating material in the future.

## 1. Introduction

Thermal interface materials (TIMs), typically composed of a polymer matrix with good wetting properties and thermally conductive fillers, are applied to the interfaces of mating components to reduce the interfacial thermal resistance. Recent studies have shown the excellent thermal conductivity, printability, and thermal stability of TIMs by introducing a multi-dimensional filler design composed of three-dimensional (3D) microscale Ag flakes, one-dimensional (1D) multi-walled carbon nanotubes (MWCNTs), and zero-dimensional (0D) Ag nanoparticles (Ag NPs) [1,2]. The employed quantities of Ag flakes (primary filler), MWCNTs, and Ag NPs (secondary filler) in the multi-dimensional filler design were 89.32, 0.85, and 2.86 wt%, respectively [2]. However, the safety-related issues of these nanomaterials used as secondary fillers regarding the human health and environment should be considered for practical industrial applications.

The toxicity of MWCNTs, one of the secondary filler materials, remains a controversial, but recently positive research results related to the safety have been reported. Cimbaluk et al. showed that the MWCNTs did not generate an apparent genotoxicity by means of single/double deoxyribonucleic acid strand breaks or clastogenic/aneugenic effects over any of the species, independent of the exposure period, using the alkaline version of the comet assay on erythrocytes and piscine micronucleus, also performed on erythrocytes [3]. Furthermore, the MWCNTs typically used in very small amounts for the multi-dimensional filler design are reported to be biodegradable [4,5]. However, Ag NPs, the other secondary filler material, are expensive and show toxicity [6,7,8,9,10,11,12]. For example, many studies have reported the respiratory system-related toxicity of Ag NPs which may be inhaled during the fabrication and application of TIMs [6,7,8,9]. Moreover, when Ag NPs are released into the aquatic environment, many aquatic organisms such as fish, daphnia, and algae can be negatively affected [10,11,12]. Therefore, the use of low-cost and innoxious metals instead of Ag as thermally conductive fillers could be an effective approach to overcome the cost and safety issues of TIMs.

Copper, an earth-abundant element, is 100 times cheaper than silver and has a high thermal conductivity (~385 W/m/K at room temperature). In addition, unlike silver, which is not biologically useful and must be removed from the body when consumed even in very small amounts, copper is likely safe at daily amounts below 10 mg [13]. Copper is an essential micronutrient for humans, required for the proper functioning of organs and metabolic processes such as infant growth, host defense mechanisms, bone strength, red and white cell maturation, iron transport, cholesterol metabolism, myocardial contractility, glucose metabolism, and brain development [14,15]. Hence, copper could be considered as a promising alternative to silver to achieve an industrially harmless useful heat-dissipating material.

In this study, we introduced an innoxious copper-based multi-dimensional filler composed of 3D microscale copper flakes, 1D MWCNTs, and 0D Cu NPs by substituting copper for silver in a multi-dimensional filler design, as demonstrated previously [1,2]. Cu NPs were synthesized by microwave irradiation of a solution of copper acetylacetonate in benzyl alcohol using a protocol similar to that reported previously [16]. The formation of Cu NPs was confirmed by ultraviolet-visible (UV-VIS) spectroscopy and X-ray diffraction (XRD). Cu NPs were then bound to MWCNTs and mixed with copper flakes and polyimide matrix to obtain a TIM paste. Subsequently, a coin sample of TIM was prepared by curing in a mold. The density (*ρ*), specific heat (*C*_p_), and cross-plane thermal diffusivity (*α*) of the copper-based TIM were measured using gas pycnometry, differential scanning calorimetry (DSC), and laser flash analysis (LFA), respectively.

## 2. Materials and Methods

### 2.1. Synthesis of Cu NPs

In a round-bottomed flask, a 50-mL reaction mixture of 367.9 mg of Cu(acac)_2_ dissolved in benzyl alcohol was exposed to microwaves at 800 W for 11 min. During the microwave exposure, the color of the solution changed from blue (0 min) to red. A colloidal solution was obtained by quenching the hot reaction mixture into 50 mL of pure benzyl alcohol. The precipitate was separated from the solution by centrifugation and washed twice with ethanol and once with diethyl ether, followed by drying under vacuum at 50 °C for 5 h; no size-selective precipitation was carried out.

### 2.2. Binding of Cu NPs with MWCNTs

First, 50 mg of MWCNTs (Nano Solution, TMC 220-05, outer diameter: 4–6 nm, length < 10 μm) were dispersed in 100 mL of ethanol by 450 W–10-min tip sonication (Sonics & Materials, VCX750; Newtown, CT, USA), and subsequently mixed with Cu NPs dissolved in ethanol by 3-h bath sonication (Lab Companion). The MWCNTs decorated with Cu NPs were then obtained by vacuum filtering with ethanol washing and drying in a vacuum oven for 12 h at room temperature. The relative concentration between the Cu NPs and MWCNTs could be controlled by changing the mass of the Cu NP powder mixed with MWCNTs.

### 2.3. Mixing of the Fillers with the Matrix and Preparation of Specimens

Cu flakes (Alfa Aesar, 1–5 µm, 99.9%, Haverhill, MA, USA) and MWCNTs coated with Cu NPs were mixed with 20-wt% polyimide (Huntsman Chemical Company, Matrimid^®^ 5218, The Woodlands, TX, USA) dissolved in 1-methyl-2-pyrrolidone (Sigma-Aldrich, 328634, 99.5%, St Louis, MO, USA) by stirring for 5 min to obtain the TIM paste. To prepare the coin specimens for the LFA, the TIM paste was poured into a Teflon mold and cured for 4 h at 240 °C. Before pouring the paste into the mold, a releasing agent (NABAKEM, FLEX-A, Pyeongtaek, Korea) was applied to the surface of the mold to easily detach the coin specimens from the mold.

### 2.4. Characterization

The cross-plane thermal diffusivity (*α*) of the coin specimen of the copper-based TIM was measured using the LFA (Netzsch, LFA-447, Selb, Bavaria, Germany), while the specific heat (*C*_p_) was measured at 10 °C/min using DSC (Mettlertoledo, DSC1 STARe System, Columbus, OH, USA) under a nitrogen atmosphere in the range of 15–40 °C. The density (*ρ*) of the coin specimen was measured with a gas pycnometer (InstruQuestInc, Humipyc model 2, Boca Raton, FL, USA) with helium gas. The cross-plane thermal conductivity (*k*) was calculated using the relation *k = ρC*_p_*α*. According to the datasheets provided by the instrument manufacturers and measurements with three specimens, the uncertainties (confidence level: 95%) in the measurements of *α*, *C*_p_, and *ρ* were 4.4, 25, and 3.4%, respectively. Hence, the total uncertainty (confidence level: 95%) in the thermal conductivity measurement was estimated to be 26%. The phase transformation of the Cu NPs on the surface of the MWCNTs was characterized using DSC under a nitrogen atmosphere with a preset temperature profile consisting of three stages: heating (10 °C/min) from 25 to 300 °C, holding at 300 °C for 1 min, and cooling (−10 °C/min) from 300 to 25 °C.

## 3. Results and Discussion

**Figure 1:**
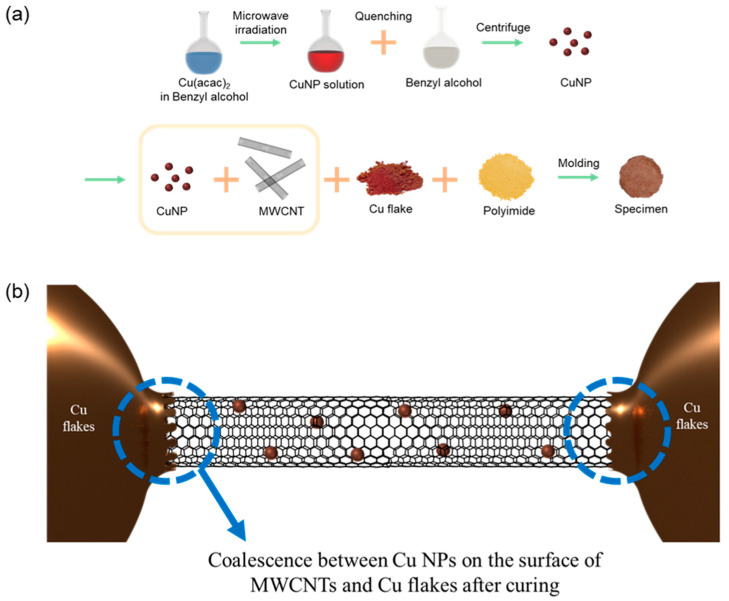
(**a**) Preparation of the copper-based thermal interface materials (TIM) and (**b**) schematic of the multi-dimensional filler design.

The copper-based TIM was prepared following the procedure illustrated in Figure 1a. Cu NPs functionalized with phenyl groups were synthesized by microwave irradiation of a solution of copper acetylacetonate in benzyl alcohol. The Cu NPs functionalized with the phenyl groups were then mixed with MWCNTs to form MWCNTs assembled with Cu NPs through π–π interactions. The MWCNTs coated with Cu NPs were mixed with Cu flakes to construct a copper-based multi-dimensional filler similar to the reported silver-based structure [1,2]. The 1D MWCNTs coated with the Cu NPs constructed effective thermal bridges between microscale copper flake islands, as shown in Figure 1b. The Cu NPs on the surface of the MWCNTs coalesced with microscale copper flakes at a curing temperature considerably below the melting temperature of the bulk Cu, which significantly enhanced the phonon transport.

**Figure 2:**
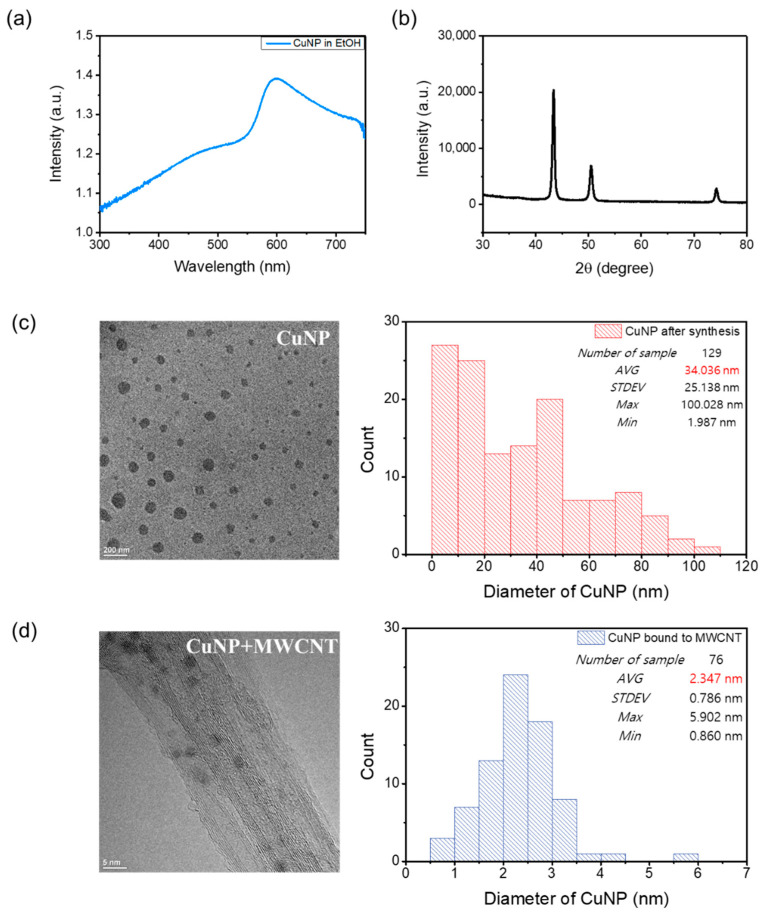
Characterization of the copper nanoparticles (Cu NPs). (**a**) Ultraviolet–visible (UV-VIS) absorption spectrum of the as-prepared Cu NP dispersion (a.u.: arbitrary unit), (**b**) X-ray diffraction (XRD) pattern of the synthesized Cu NPs, (**c**) transmission electron microscopy (TEM) image and size distribution of the as-synthesized Cu NPs, and (**d**) TEM image and size distribution of the Cu NPs bound to multi-walled carbon nanotubes (MWCNTs).

**Figure 3:**
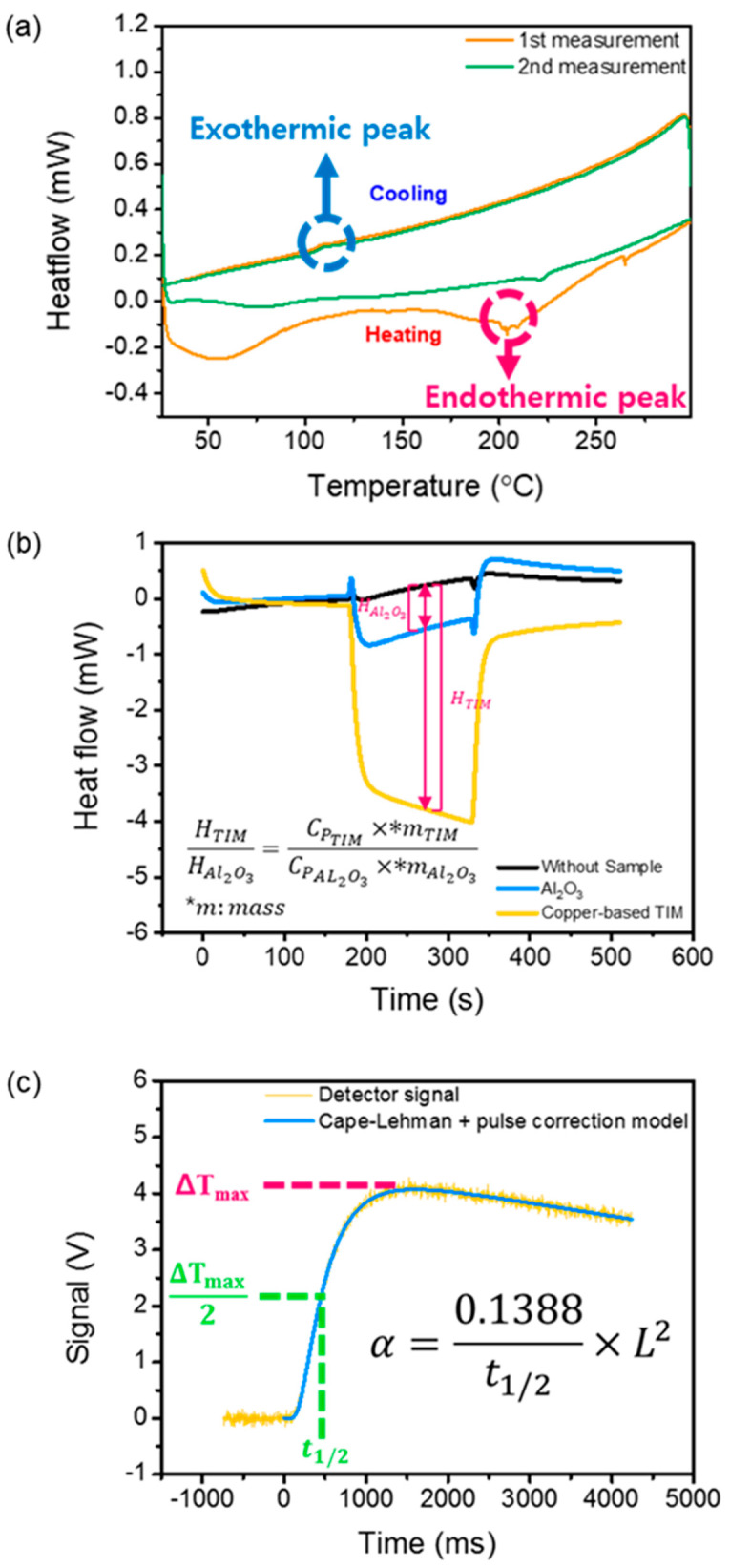
Thermal characterization of the Cu-based materials. (**a**) Differential scanning calorimetry (DSC) analysis of the phase change of the Cu NPs, (**b**) DSC analysis for the measurement of the specific heat (*C*_p_), and (**c**) laser flash analysis (LFA) for the cross-plane thermal diffusivity (*α*) of the Cu-based TIM.

At room temperature, a solution of Cu(acac)_2_ in benzyl alcohol is blue. Upon exposure to microwaves at 800 W for 1 min, the color changed to green, indicating a transition to partly reduced small clusters or NPs. Further irradiation of the solution for 10 min led to the formation of a deep-red liquid, which indicates the formation of copper NPs. The UV-VIS absorption spectrum of the solution after the microwave irradiation is shown in Figure 2a. The characteristic absorption peak around 575 nm is attributed to the surface plasmon band of nonoxidized Cu NPs [17,18]. The broadness of the absorption band originates from the wide size distribution of the copper NPs. Therefore, the monodispersity can be still improved and further size tuning of NPs can be carried out. The XRD analysis confirmed that the Cu NPs were successfully prepared, as shown in Figure 2b. The sharp peaks at 2θ of approximately 43.3, 50.5, and 74.2° indicate that the synthesized Cu NPs were well crystallized [19,20]; no characteristic peaks of Cu oxides were detected. To evaluate the size distribution of Cu NPs, a transmission electron microscopy analysis was carried out. Figure 2c shows that the size of the NPs was in the range of ~2 to 100 nm with an average of 34 nm. The size distribution of the Cu NPs was also evaluated after binding with MWCNTs, as shown in Figure 2d. Notably, the analysis results show that MWCNTs bound to small NPs with an average size of approximately 2.3 nm. As the melting point of the Cu NPs decreases with the decrease in the particle size because of their unstable atoms and large surface area, the selective binding between MWCNTs and small Cu NPs reduces the curing temperature (melting point of Cu NPs bound to MWCNTs) of the TIM [21]. The phase change of the Cu NPs was investigated using DSC with two cycles, as shown in Figure 3a. An endothermic valley and exothermic peak were observed during the first heating and cooling of the Cu NPs, respectively. The endothermic peak at approximately 200 °C is attributed to the melting of the Cu NPs. The reaction was not reversible and was completed within the first cycle. In the second cycle, the endothermic peak was shifted to a higher temperature, implying aggregation of Cu NPs. Hence, the coalescence between Cu NPs and Cu flakes occurred at approximately 200 °C, significantly lower than the melting temperature of bulk copper (1085 °C).

Figure 3b,c show the results of the DSC for the specific heat and LFA for the cross-plane thermal diffusivity necessary for the thermal conductivity measurement, respectively. The specific heat measurements were carried out through a standard procedure using a sapphire reference. Three measurements are necessary to obtain the specific heat. First, a baseline was recorded (black line). This is the response for the empty crucible, yielding a signal bias inherent in the system. Second, a reference measurement (blue line) was carried out, in which sapphire with a well-known specific heat was analyzed for comparison with a test sample. Third, the copper-based TIM was tested (yellow line). The baseline allows removal of system bias from the data, while the reference test enables calculation of the specific heat of the copper-based TIM based on the ratio of the specific heat of the TIM to that of the sapphire. The laser flash method, proposed by Parker, was used to determine the cross-plane thermal diffusivity of the copper-based TIM [22]. The method uses a short laser pulse to heat the front surface of a thin disk-shaped specimen. An infrared detector records the transient voltage change corresponding to the rear surface temperature. We used the voltage change to obtain the thermal diffusivity *α* using the relation *α* = 0.1388 L^2^/t_1/2_, where L is the thickness of the sample and t_1/2_ is the time required for the temperature to increase to half of its maximum value. We used the most sophisticated model (blue line) established by Cape and Lehman, which considers finite pulse effects as well as facial and radial heat losses [23].

**Figure 4:**
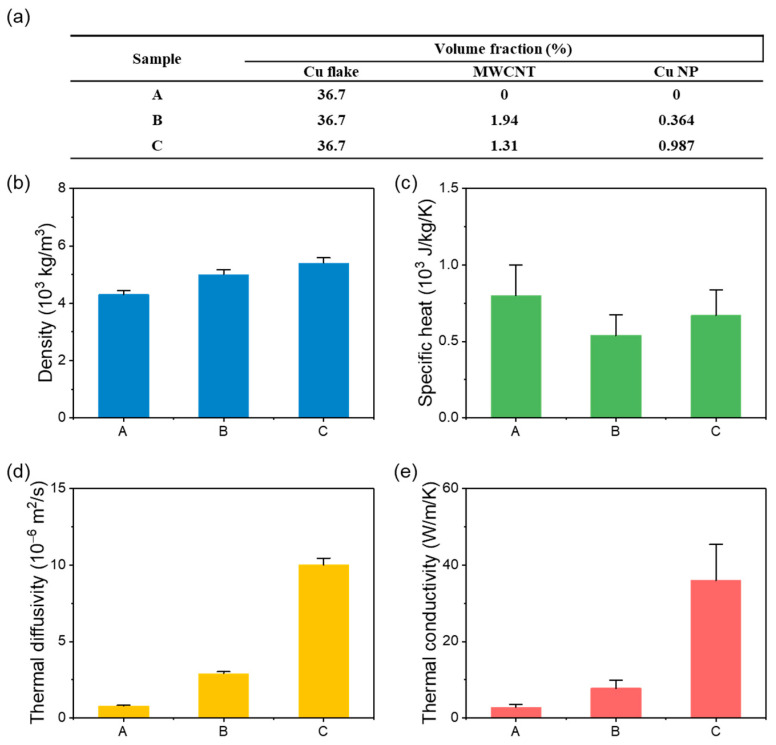
Thermal characterization. (**a**) TIM sample characteristics. (**b**) Densities, (**c**) specific heats, (**d**) thermal diffusivities, and (**e**) thermal conductivities of the sample TIMs.

Parametric studies were performed to verify the effect of the multi-dimensional filler, as shown in Figure 4. The volume fraction of the Cu flakes was fixed at 36.7 vol%, according to the previous study on a Ag-based multi-dimensional filler design, while the contents of MWCNTs and Ag NPs were varied [1]. With the increase in the copper content, *ρ* of the TIM increased (Figure 4b), while its *C*_p_ decreased (Figure 4c). The increase in *ρ* and decrease in *C*_p_ due to the addition of the MWCNTs functionalized with the Cu NPs cancelled out. However, *α* was significantly affected by the small change in the filler content, as shown in Figure 4d. The similar trends of *α* (Figure 4d) and k (Figure 4e) show that the change in k is dominated by the change in *α*. As a control, only Cu flakes were employed as fillers to investigate the thermal properties of a TIM without the multi-dimensional fillers (sample A). k of sample A was 1.7 W/m/K. Despite the high intrinsic k of copper, the low k of the Cu-flake TIM could be attributed to the poor connection among the fillers. When the multi-dimensional design with filler contents (MWCNT: 1.313 vol%, Cu NPs: 0.987 vol%) optimized in the previous study was used (sample C), the thermal conductivity reached 36 W/m/K [1]. The improvement of the k in the sample C is resulted from the formation of the thermal percolation network of the fillers. The result shows that using the multi-dimensional fillers is effective in paving the thermal percolation network by the synergistic effect which is a phenomenon that a combination of fillers with different dimensions can efficiently build the percolation network in a composite [24,25]. We also checked whether k decreased when the volume content of the fillers deviated from the optimized filler contents (MWCNTs: 1.313 vol%, Cu NPs: 0.987 vol%). When the volume fraction of the Cu NPs was decreased to 0.364 vol% and the content of the MWCNTs was increased to 1.936 vol% (sample B), k was considerably reduced (7.8 W/m/K), confirming the important role of the Cu NPs covering the MWCNTs for the construction of phonon pathways at the junctions between the Cu flakes and MWCNTs. In addition, the multi-dimensional filler design is applicable when any metal is used for the 3D and 0D fillers. Compared to k (4.722 W/m/K for a filler content of 57 vol%) of a recently reported copper-based TIM, the present copper-based TIM with multi-dimensional fillers exhibits a considerably higher k even at a lower volume fraction of fillers [26]. In addition, the thermal conductivity of our copper-based TIM (36 W/m/K) is much higher than those of commercial TIMs (0.39–8.7 W/m/K) [27,28]. Thus, the multi-dimensional filler design bridging the 3D fillers with lower-dimensional fillers including the MWCNTs and Cu NPs could save expensive filler materials and provide an excellent thermal conduction at the interfaces.

Although the introduction of low-dimensional fillers may raise concerns about safety issues, recent research works on the toxicity of MWCNTs and Cu NPs indicate that the concern is not an issue to be seriously considered. Moreover, replacement of Ag NPs with Cu NPs proposed in this study is an effective approach toward highly conductive and affordable TIMs urgently needed in the industry.

## 4. Conclusions

We demonstrated a safe low-cost copper-based TIM with a high thermal conductivity by introducing a multi-dimensional filler design bridging the 3D fillers with lower-dimensional fillers including the MWCNTs and Cu NPs. We confirmed that copper could be a promising alternative to silver in realizing highly conductive TIMs. The cross-plane thermal conductivity of the copper-based TIM reached 36 W/m/K. Considering its innocuousness, high thermal conductivity, and low cost, the copper-based TIM could save expensive filler materials and provide an excellent thermal conduction at interfaces. Thus, it could be used as an industrially harmless and useful thermal management material in the future.

## Data Availability

The article contains complete data used to support the findings of this study.
